# Thermo-Rheological and Shape Memory Properties of Block and Random Copolymers of Lactide and ε-Caprolactone

**DOI:** 10.3390/polym13040627

**Published:** 2021-02-19

**Authors:** Marco Naddeo, Andrea Sorrentino, Daniela Pappalardo

**Affiliations:** 1 Dipartimento di Scienze e Tecnologie, Università del Sannio, via de Sanctis snc, 82100 Benevento, Italy; marco.naddeo@unisannio.it; 2 Institute for Polymers, Composites and Biomaterials (IPCB-CNR), P.le E Fermi, 1, 80055 Portici, Italy

**Keywords:** block copolymers, lactide, caprolactone, thermal properties, rheological properties, shape memory

## Abstract

Biodegradable block and random copolymers have attracted numerous research interests in different areas, due to their capability to provide a broad range of properties. In this paper, an efficient strategy has been reported for preparing biodegradable PCL-PLA copolymers with improved thermal, mechanical and rheological properties. Two block-copolymers are synthesized by sequential addition of the cyclic esters lactide (L-LA or D,L-LA) and ε-caprolactone (CL) in presence of a dimethyl(salicylaldiminato)aluminium compound. The random copolymer of L-LA and CL was synthetized by using the same catalyst. Chain structure, molar mass, thermal, rheological and mechanical properties are characterized by NMR, SEC, TGA, DSC, Rheometry and DMTA. Experimental results show that by changing the stereochemistry and monomer distribution of the copolymers it is possible to obtain a variety of properties. Promising shape-memory properties are also observed in the di-block copolymers characterized by the co-crystallization of CL and L-LA segments. These materials show great potential to substitute oil-based polymers for packaging, electronics, and medicine applications.

## 1. Introduction

Oil-derived polymers still dominate the market today, due to their low cost, high availability, and durability. However, the problem related to their end-of-life disposal presents an environmental challenge that is no longer sustainable in the long term [1,2]. In recent years, constant scientific efforts have been dedicated to the production of synthetic biodegradable polymers with a reduced environmental impact [3]. Despite the huge amount of research efforts, the number of high-performance biodegradable polymers commercially available is still very limited. Among these, linear aliphatic polyesters represent the class of biodegradable materials that are the most studied [4]. Among these polymers, the leading position is undoubtedly held by poly(lactide) (PLA) and poly(ε-caprolactone) (PCL). Their synthesis can be achieved by the ring-opening polymerization (ROP) of the cyclic esters lactide (LA) and ε-caprolactone (CL), respectively [4,5]. Both PLA and PCL have found applications in various sectors such as biomedical and packaging, thanks to their good properties [3,6]. Among other things, they are biocompatible and resorbable and their degradation products are metabolized during the Krebs cycle [6,7]. PLA can be derived from several renewable resources such as the fermentation products of starch [8]. This possibility has placed PLA at the forefront of the emerging biodegradable plastic industry, including in the context of a circular economy. The chirality of lactic acid gives rise to three lactides: L-, D-, and meso-lactide, whose polymerization will produce PLA of various stereoregularities. Homochiral isotactic PLA (either PLLA or PDLA) shows a melting transition of 180 °C and a glass transition in the range 55–60 °C [9]. The ROP of racemic mixture of D and L lactide (D,L-LA or rac-LA), in the absence of a stereoselective catalysts, will produce instead an amorphous polymer, the poly-(D,L-lactide), with a glass transition temperature close to 50 °C [10]. Unfortunately, the brittle nature of isotactic PLA limits its application [11]. For instance, for biomedical applications, because the glass transition temperature of poly-(D,L-lactide) (PDLLA) and poly(L-lactide) (PLLA) is well above the human body temperature, these materials are too stiff [6].

On the contrary, the poly(ε-caprolactone) (PCL) is in the rubbery state at room temperature, showing a glass transition temperature at about −60 °C and a melting point at about 65 °C [12]. The PCL has been exploited in the biomedical field to develop long-term delivery system or scaffolds for bone tissue engineering [13,14]. At variance with PLA, the PCL is a highly processable polymer, highly hydrophobic, with a degradation time of about two years [15].

The complementary characteristics of PLA and PCL have suggested taking advantage of both through the copolymerization of the respective monomers, lactide (LA) and ε-caprolactone (CL), in various compositions.

The synthesis of CL/LA copolymers has been widely investigated in recent years, focusing on either block or random copolymers [16,17,18,19,20]. Copolymers of CL and LA generally show softer character in comparison with the PLA and PCL homo-polymers. Moreover, the hydrolytic degradation rate of the copolymers of LA and CL is strongly influenced by the composition, as a consequence of the degree of crystallinity and the hydrophobicity. It was found that the degradation rate increase by increasing the LA amount and by the degree of randomness in the case of CL/LA copolymers [21]. The final properties of the copolymers are dictated not only by their composition. Additionally, the details of the macromolecular structure, such as the molecular weight and dispersity, kind of end-groups [22], the polymer microstructure and architecture are important factors [23]. Synthetic strategies have therefore been used to control the design of the polymeric chains. Several research efforts have focused on the synthesis of well-defined LA/CL copolymeric chain, either block [17,18,20,24], or random [19,25,26,27,28].

Pappalardo et al. [20,29] have described dimethyl(salicylaldiminato)aluminium compounds in the ring-opening polymerization of caprolactone and lactides disclosing a well-controlled polymerization with absence of transesterification reaction and a certain living behavior of the polymerization, when the phenyl substituent on the phenoxyimine ligand of the catalyst was perfluorurated.

This family of catalysts were highly versatile and active not only in the ROP of traditional cyclic esters i.e., glycolide, lactide and caprolactone [30,31], but also oflarge ring macrolactone [32,33] and ofnovel synthetized thiol-functionalized lactide [34,35]. Block and random copolymers have been obtained with this class of catalyst. By sequential addition of the two monomers, first the L-LA (or the D,L-LA) and then the CL, di-block copolymers poly-(L-lactide-*block*-ε-caprolactone) and poly(D,L-lactide-*block*-ε-caprolactone) were obtained. The same class of catalysts was able to copolymerize ε-CL with L-LA and ε-CL with D,L-LA in a random fashion, in the absence of transesterification reactions [20]. Notably, the copolymerization reactions were better controlled than those carried out in the presence of other catalysts, i.e., SnOct_2_, where the transesterification were responsible of the randomized structure with enlargement of the polymer dispersities.

Previous studies have highlighted that not only the composition, but also the architecture and monomers distributions of copolymers could strongly affect their thermal, mechanical and aggregation properties [36,37,38].

In this work, PCL-PLA copolymers are prepared with identical composition and analogous molecular weights and dispersities, but differing for stereochemistry and monomer distribution. Two di-block copolymers, namely poly-(L-lactide-*block*-ε-caprolactone) and poly(D,L-lactide-*block*-ε-caprolactone) and a random poly-(L-lactide-*co*-ε-caprolactone) were successfully obtained. Their thermal and rheological properties are found strongly dependent by the molecular structures. Crystalline domains with different melting temperature are formed and utilized as reversible crosslinking in the polymer network. It has opened the way to a novel class of thermo-responsive shape memory polymers with programmable properties.

## 2. Materials and Methods

Moisture and air-sensitive materials were manipulated under nitrogen using Schlenk or glovebox techniques. Toluene was refluxed over sodium/benzophenone and distilled under nitrogen prior to use. Benzyl alcohol (purchased from Sigma Aldrich) was distilled from CaH_2_ and stored on 4 Å molecular sieves. Other solvents were purchased from Sigma Aldrich and used as received. ε-caprolactone was distilled in vacuum from CaH_2_ and stored on 4 Å molecular sieves. D,L-lactide and L-lactide were recrystallized twice from dry toluene, and then were dried in vacuo over phosphorus pentoxide for 72 h. The dimethyl(salicylaldiminato)aluminium compound (**I**) was synthesized according to a previously reported procedure [39].

Commercial Polylactic Acid (PLA) was supplied by Nature Works (NatureWorks^®^ PLA Polymer 2002D, Mw = 200,000–220,000 by GPC, density 1.24 g/mL at 25 °C) in pellet form. Polycaprolactone was supplied by Sigma Aldrich (Mn = 70,000–90,000 by GPC, Mw/Mn < 2, density 1.145 g/mL at 25 °C).

### 2.1. Material Characterization

NMR spectra of polymers were obtained in CDCl_3_ at room temperature on Bruker Avance 300 and 400 spectrometers (^1^H: 400 MHz; ^13^C: 100 MHz and ^1^H: 300 MHz; ^13^C: 75 MHz, respectively). The resonances and coupling constants are reported in ppm (δ) and Hz (J), respectively. ^1^H NMR spectra were referenced to the residual solvent proton at δ 7.26 ppm; ^13^C NMR spectra were referenced to the ^13^C signal of CDCl_3_ at δ 77.16 ppm. Spectra were recorded on Bruker TopSpin v2.1 software. Data processing was performed using MestReNova v9.0.0 software.

Molecular weights (Mn and Mw) and dispersities (*Ð*) were measured by size exclusion chromatography (SEC). The measurements were performed at 30 °C on a Waters 1525 binary system equipped with a system of four Styragel HR columns (7.8 × 300 mm^2^; range 103–106 Å), Waters 2414 Refractive Index (RI) detector and a Waters 2487 Dual λ Absorption (UV, λ abs = 220 nm) detector. Tetrahydrofuran was used as eluent at a flow rate of 1.0 mL/min. Narrow polystyrene standards were used as reference and data processing was performed using Waters Breeze v3.30 software.

The thermal characterization was performed by dynamic Differential Scanning Calorimetry (DSC) analysis and by thermogravimetric analysis (TGA). The TGA measurements were carried out using a Mettler Toledo TGA30 analyzer. The experiments were performed using about 10 mg of sample from room temperature to 600 °C at 10 °C/min under nitrogen atmosphere (60 mL/min). The degradation onset temperature (T5%) was determined as the temperature at the 5% of mass loss. The first derivative of the TGA curve (DTG) was utilized for analyses the various decomposition steps.

For DSC measurements a Mettler Toledo DSC822e instrument was used. Thermal cycles was composed by three cycles: a first heating scan, from −50 °C to 200 °C, to analyze the thermal history of the sample, a cooling scan from 200 °C to −50 °C to analyze their crystallization behavior from the melt state and, finally, another heating scan from −50 °C to 200 °C to investigate their melting/recrystallization behavior. The scans were performed with a heating rate of 10 °C/min run under nitrogen purge (20 mL/min). The degree of crystallinity (*Xc*) was determined by means of the following equation:

(1) $$X_{c}=\frac{\Delta H_{m}}{\Delta H_{f}}*100\%$$

where Δ*Hf* is the enthalpy value of a pure crystalline material, Δ*Hm* is the enthalpy corresponding to the fusion process. The reference value taken for Δ*Hf* of PCL and PLA was 139.5 J/g [40] and 93 J/g [41,42], respectively.

Thermo-rheological characterizations of the samples were performed in a HAAKE MARS III rheometer with 25mm diameter parallel-plate geometry. The test samples were manually pressed into 1 mm thick plates at ambient temperature and then heated and pressed between the plates rheometer up to reach a gap of 0.5 mm. For each polymer, the linear viscoelastic behavior and dynamic modulus (G’ and G″ against f) were determined in oscillatory tests at different temperatures. Additionally, temperature sweep measurements were also carried out at a frequency of 1 Hz and a strain of 0.1% from 80 °C to 200 °C in order to elucidate the eventual presence of any transition temperature.

Shape memory ability was analyzed under tensile deformation with a DMA Q800 (TA Instruments) in a sequence of strain-stress controlled mode. The powder samples were molded in a Carver laboratory press, at the temperature of 180 °C, 10 bar, using a Collin P200E laboratory press. Films 100–110 µm thick were quickly cooled in an ice bath and then stored in dry conditions for 1 week before each characterization tests. The sample was pre-heated to a deformation temperature (Td), and then elongated up to a constant strain (ε_d_) at a strain rate of 10 mm/min. The stress reached was then held constant while the sample was cooled down to 20 °C at 3 °C/min and kept for 15 min at this temperature. The force applied was then lowered to the minimum (0.001 N) for follow the length recovery and the sample allowed to stabilize for 5 min in isothermal. Finally, the sample was heated back to the deformation temperature (Td) at 3 °C/min, while the shape recovery was monitored. The recovery and Fixity ratio (Rr and Rc) are generally used for characterize the shape memory ability of a material. In particular, Rc indicates the ability of the material to hold the imposed strain in the stress-free state (ratio between strain after and before stress removal at 20 °C), while Rr indicates its ability to recovery its initial shape (ratio between strain after and before sample heating at Td) [43].

### 2.2. Material Synthesis

#### 2.2.1. Synthesis of Poly(L-lactide-*block*-ε-caprolactone) (Block-L)

Into the glovebox, a 100 mL round bottom flask was filled sequentially with L-lactide 0.70 g (4.8 mmol), complex (**I**) 0.020 g (0.050 mmol), 0.50 mL of a solution of benzyl alcohol (0.1 M, 0.050 mmol) and 3.0 mL of dry toluene. The reaction mixture was thermostated at 90 °C for 72 h. After the indicated time, an aliquot (0.5 mL) of the polymerization mixture was quenched in hexane, and the polymer was recovered by filtration, dried in vacuo, and analyzed by GPC (Mn = 7.6 kDa; *Ð* = 2.7). Then, ε-caprolactone (1.5 mL, 13.5 mmol) was added to the residual mixture and a final volume of 10 mL was reached by the addition of dry toluene. The reaction mixture was left at the temperature of 90 °C for 24 h. The reaction was quenched in cold hexane and the polymer purified by dissolving it in CH_2_Cl_2_ and precipitating from rapidly stirring methanol. The polymer was then recovered by filtration and dried at 40 °C in a vacuum oven.

^1^H NMR (CDCl_3_, 25 °C): δ = 1.37 (m, 2H, -CH_2_-), 1.56 (d, 6H, -CHCH_3_-), 1.62 (m, 4H, -CH2-), 2.30 (t, 2H,-CH_2_C(O)O-), 4.06 (t, 2H, -CH_2_OC(O)-), 5.17 (q, 2H, -_CHCH3_-). Yield = 1.70 g (76% *w*/*w*). GPC: Mn = 43.4 kDa; *Ð* = 1.4.

#### 2.2.2. Synthesis of Poly(D,L-lactide-*block*-ε-caprolactone) (Block-D,L)

The copolymer was prepared as above, but D,L-lactide was used instead of L-lactide. To check the molecular weight of the first poly(D,L-lactide) block, after 72 h of reaction, an aliquot (0.5 mL) of the polymerization mixture was quenched in hexane, and the polymer was recovered by filtration, dried in vacuo, and analyzed by GPC (Mn = 16.6 kDa; *Ð* = 1.3). Subsequently ε-caprolactone (1.5 mL, 13.5 mmol) was added and the same procedure for the preparation of the above copolymer was followed.

^1^H NMR (CDCl_3_, 25 °C): δ = 1.37 (m, 2H, -CH2-), 1.59 (m, 6H,-CHCH_3_-), 1.65 (m, 4H,-CH_2_-), 2.30 (m, 2H, -CH_2_C(O)O-), 4.05 (m, 2H, -CH_2_OC(O)-), 5.14 (m, 2H, -CHCH_3_-).Yield = 1.98 g (88% *w*/*w*). GPC: Mn = 54.7 kDa; *Ð* = 1.4.

#### 2.2.3. Synthesis of Poly(L-lactide-*co*-ε-caprolactone) (Ran)

The copolymer was prepared as follow. Into the glovebox under nitrogen atmosphere, a 100 mL round bottom flask was filled sequentially with L-lactide 0.70 g (4.8 mmol), ε-caprolactone (1.5 mL, 13.5 mmol), complex (**I**) 0.020 g (0.050 mmol), 0.50 mL of a solution of benzyl alcohol (0.1 M, 0.050 mmol) and dry toluene to reach a final volume of 10 mL. The reaction mixture was thermostated at 90 °C for 72 h. After the indicated time, the reaction was quenched in cold hexane and the polymer purified by dissolving in CH_2_Cl_2_ and precipitating from rapidly stirring in methanol. The polymer was recovered by filtration, dried at 40 °C in a vacuum oven. ^1^H NMR (CDCl_3_, 25 °C): δ = 1.38 (m, 2H,-CH_2_-), 1.52 (m, 6H,-CHCH_3_-), 1.64 (m, 4H,-CH_2_-), 2.30–2.40 (m, 2H,-CH_2_C(O)O-), 4.05–4.13 (m, 2H,-CH_2_OC(O)-), 5.12 (m, 2H, -CHCH_3_-).Yield = 90% GPC: Mn = 71.8 kDa; *Ð* = 1.51

## 3. Results

### 3.1. Synthesis of Block and Random LA/CL Copolymers

The synthesis of the copolymers was carried out in the presence of the dimethyl(salicylaldiminato)aluminium compound (I) activated by benzyl alcohol in toluene solution, at 90 °C.

**Scheme 1:**
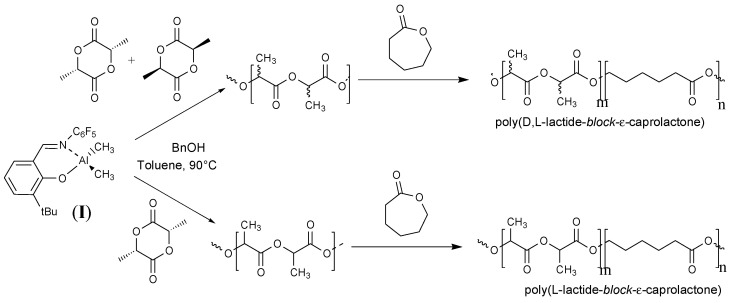
Synthesis of LA/CL block copolymers.

The block copolymers were prepared by sequential addition of the two monomers, first the lactide (L-LA or rac-LA) and then the CL (Scheme 1). The feed molar ratio of CL:LA of the block copolymers was settled to be 3:1. As a consequence of the living character of the ROP polymerization in the presence of catalyst I, the di-block copolymers poly-(L-lactide-block-ε-caprolactone) and poly(rac-lactide-block-ε-caprolactone) were successfully obtained. The ROP of L-LA, as well as in that of D,L-LA, were monitored by 1H NMR, to determine the conversion of lactide. When the lactide reached almost full conversion, a sample was withdrawn from the polymerization mixture and it was analyzed by GPC, showing monomodal distribution. Then, ε-caprolactone monomer was added to the reaction mixture and allowed to react for the selected time, to give the block copolymer PLA-PCL. The formation of the block copolymers was confirmed by the increasing of the molecular weight of the copolymer, with respect to the molecular weight of the PLA first block, while the GPC curve preserved a monomodal behavior (see below).

Notably, this is a quite uncommon behavior: traditionally, for instance with aluminum isopropoxide, the di-block PLA-PCL copolymers have been only achieved via a CL first route [18].

A random copolymer of LLA and Cl (Table 1, sample 3) was prepared by mixing in the two monomers in the same proportion (LLA:CL = 30:70) used for the block copolymers. Even in this case the polymerization was carried out in toluene solution, at 90 °C, by using 1 equiv of BnOH as initiator.

**Scheme 2:**
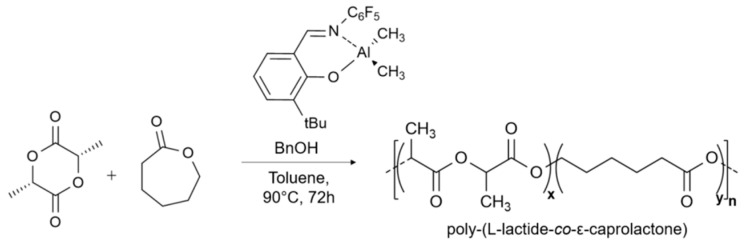
Synthesis of Random LLA/CL Copolymer.

The synthesis of the random copolymer is illustrated Scheme 2.

The block and random copolymers were fully characterized by ^1^H and ^13^C NMR, GPC and DSC analyses. The data of copolymers composition, molecular weight and their distribution are reported in Table 1.

The relative contents of each monomer in the copolymers were determined by ^1^H NMR spectroscopy in CDCl_3_, through the ratio of the integrated values of the methylene signals of the ε-CL units around 4.0 ppm and the methine signals of LA around 5.2 ppm. The copolymer composition parallels the ε-CL/LA molar ratio in the feed (see Table 1), thus indicating a good control in the polymerization. This behavior is observed both in the block and random copolymerization.

**Figure 1:**
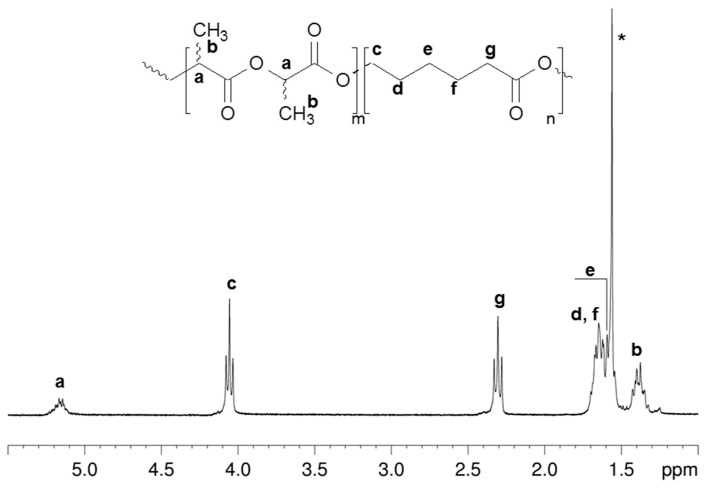
^1^H NMR spectrum of poly-(D,L-Lactide-*block*-ε-caprolactone) (CDCl3, 25 °C, 400 MHz). * Residual water in deuterated solvent.

**Figure 2:**
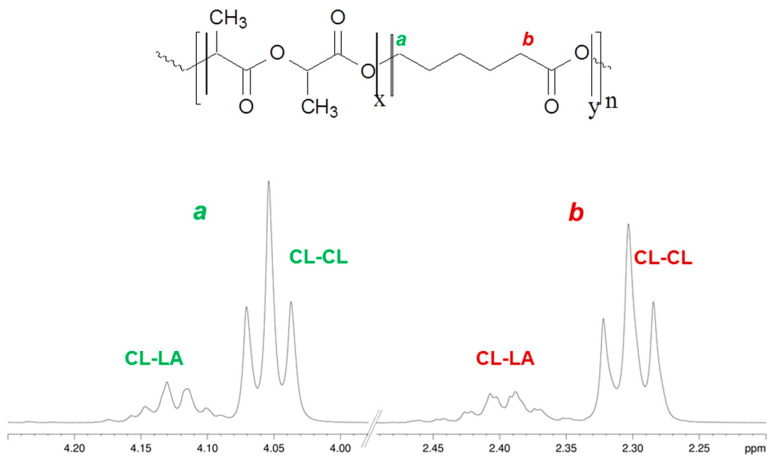
^1^H NMR spectrum of poly-(L-Lactide-*co*-ε-caprolactone) (CDCl_3_, 25 °C, 300 MHz) in the methylene regions.

The microstructure characterization of the copolymers was carried out by ^1^H and ^13^NMR spectroscopy. When comparing the ^1^H NMR spectra, while for the block copolymers poly-(L-lactide-*block*-ε-caprolactone) and poly(D,L-lactide-*block*-ε-caprolactone) the signals observed were just those relative to the homosequences, i.e., those that one could observe by the superimposition of the corresponding homopolymers NMR spectra (see e.g., Figure 1), in the case of the random copolymer poly(L-lactide-*co*-ε-caprolactone) additional resonances, attributable to the CL-LA heterosequences, were instead observed. In particular, the caprolactone methylene signals in α and ε positions respect to the carbonyl are sensitive to their surroundings, and appeared at lower fields when the next unit is a lactide (Figure 2).

By comparing, in the ^1^H NMR spectrum, the intensity of the signals of the caprolactone methylene protons in α and ε position respect to the carbonyl (-CH_2_-C=O and -COOCH_2_-) of the CL-LA heterosequences with the same methylene protons for the CL-CL homosequences, it is possible to evaluate the amount of heterosequences. In particular, in this latter case, the percentage of CL−LA heterodiads was 24%.

**Figure 3:**
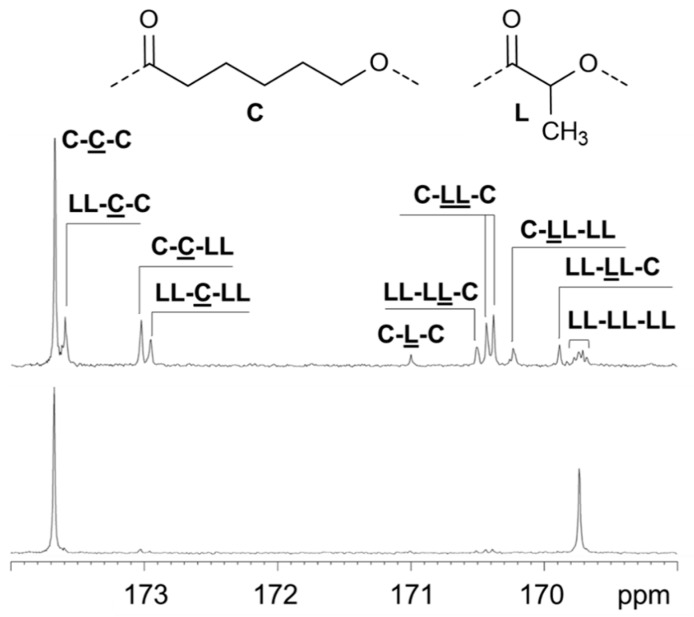
Carbonyl regions of the ^13^C NMR spectra of: (top) poly-(L-Lactide-*co*-ε-caprolactone) and (bottom) poly-(L-lactide-*block*-ε-caprolactone) (CDCl3, 25 °C, 600 MHz).

Further validations of the microstructure were derived from the analysis of the ^13^C NMR spectra of the block and random copolymers. In the ^13^C NMR spectra, indeed, diagnostic signals are the carbonyl ones, which are very sensitive to their chemical surroundings. In the case of the block copolymers just two signals relative to the CL-CL and LA-LA homosequences were observed (Figure 3). On the contrary, for the random copolymer, as expected for the case of a binary copolymerization, all the eight triads relative to the possible sequences were observed (Figure 3).

The attributions of the signals were performed accordingly to the literature. Thus, the four peaks between 173.02 and 173.74 ppm were derived from the caproyl unit, while the peaks between 170.57 and 169.54 ppm can be attributed to the lactidyl unit [16]. The signals at 171.0 ppm is attributed to the presence of single lactic unit, flanked by two caprolactones, and can be derived by the occurrence of transesterification reactions, which can become more frequent at higher temperature [20]. From these data, it is possible to evaluate the average sequences blocks lengths [16], which were LLA = 1.2 and LCL = 4.7. The expected average sequence block lengths calculated in the case of a Bernouillian statistics [44], and assuming that the lactide does not trans-esterify to single lactyl, are LLA(*th*) = 1.28 and LCL(*th*) = 4.54. These values nicely fit with the experimental average sequential blocks lengths, and further confirm the copolymer random microstructure. The slight difference between the experimental and calculated values could be due to the occurrence of some transesterification reactions.

### 3.2. Thermogravimetric Analysis

**Figure 4:**
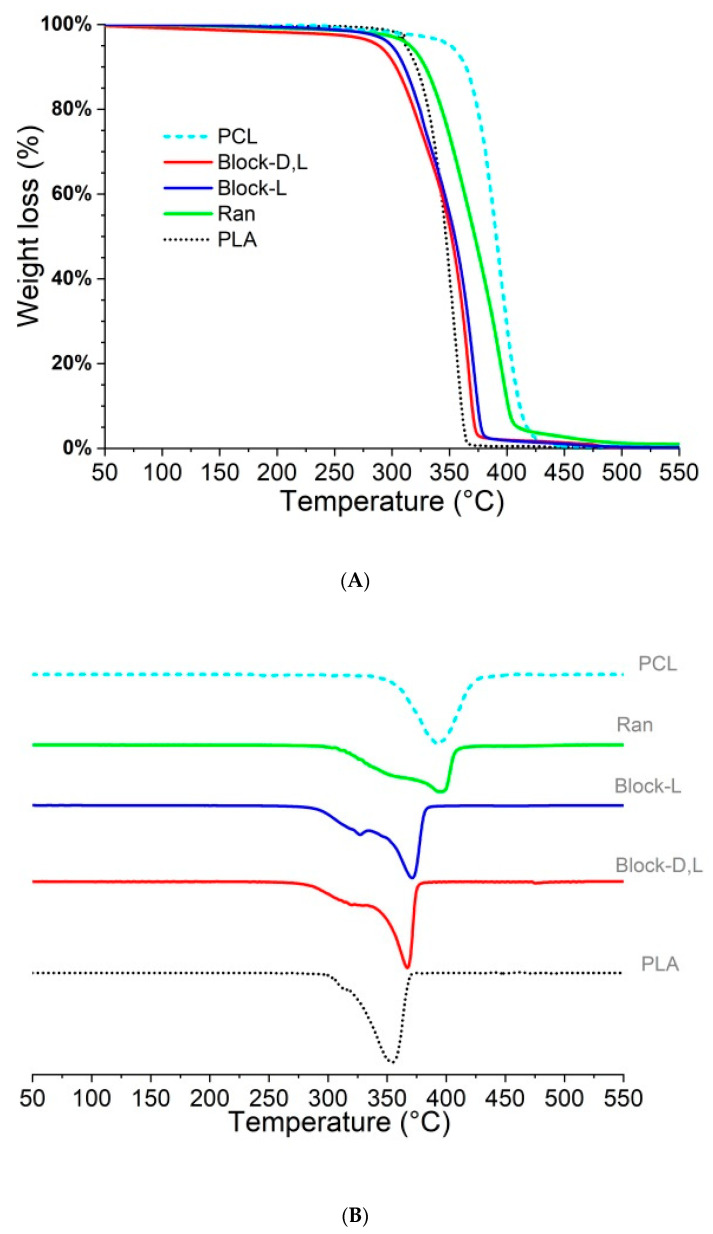
TGA curves (**A**) and DTG curves (**B**) of the analyzed samples.

Thermogravimetric analysis (TGA) has been carried out in order to both determine the thermal stability of the samples and verify the presence of impurity such as catalytic residue, solvents or monomers. Figure 4A reports the weight loss of the synthetized materials as function of the temperature in nitrogen atmosphere. For comparison, degradation curves for commercial PLA and PCL are also reported. At higher temperatures, all curves show a very small amount of inert residue (less than 0.1%). It suggests the absence of inorganic impurity in the synthetized materials. As expected, the PLA degrade at temperatures lower than that of PCL [45,46]. In particular, the temperatures at 5% mass loss (T5%) were found at 312 °C and 355 °C for PLA and PCL, respectively. Both homo-polymers degrade in a single step with inflection point at 355 °C and 393 °C, respectively. The degradation curves of the synthetized block co-polymers fall between the two previous curves. However, multiple degradation steps are evident from their shapes. For a better visualization, the first derivatives of the TG curves (DTG) are reported in Figure 4B. Two peaks corresponding to the maximum degradation temperature of PLA and PCL are showed by the three co-polymers. Their position and relative high are evidently correlated to the ε-CL/LA molar ratio present in the copolymers.

For all samples, the temperatures at 5% mass loss (T5%), the peaks positions (Td1 and Td2) of the DTG curves and the temperature degradation range (ΔT1 and ΔT2) are summarized in Table 2. In particular, the degradation ranges (ΔT1 and ΔT2) are calculated as the difference between the peak position temperature (Td1 and Td2) and T5%. At constant heating rate, ΔT1 and ΔT2 are proportional to the degradation rate of the LA and ε-CL segments, respectively.

T5% of the copolymers is significantly lower than that of the homo-polymer. It could be due to the lower molar mass and higher number of end groups of the copolymers. The higher ΔT2 values showed by the co-polymers suggest a possible change of the degradation mechanism associated to the ε-CL segments. In general, it can be noticed that the synthetized samples have a thermal stability similar to that of the homo-polymers in all the degradation stages.

### 3.3. Differential Thermal Analysis

**Figure 5:**
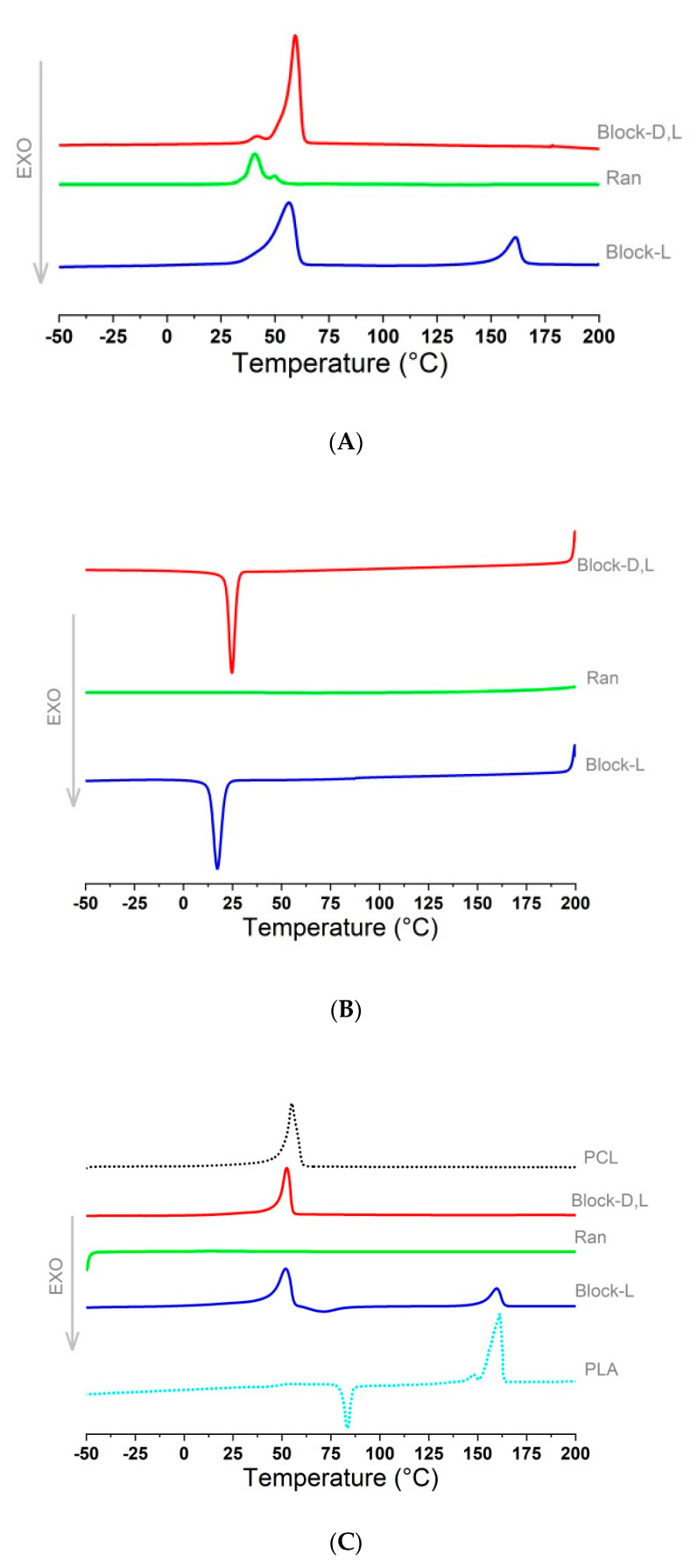
Differential scanning calorimetry (DSC) curves of the analyzed samples. (**A**) First heating; (**B**) Cooling from the melt; (**C**) Second heating.

Dynamic DSC measurements have been performed to check the thermal properties and the melt/crystallization behavior of the copolymers. In Figure 5, the results for the thermal cycles (heating–cooling–heating) performed on all the samples are presented. The first heating reported in Figure 5A shows the thermal behavior of the copolymers as obtained by synthesis: all samples show endothermic peaks indicative of their semi-crystalline morphology. Only the Block-L sample presents two melting peaks with maxima at 60 °C and 160 °C. Small exothermic peak due to cold crystallization is also observed in the first heating scan, indicating that this sample did not reach the fully degree of crystallinity after synthesis.

In Figure 5B, the cooling scans from the melt state of the copolymer are reported. It is worth noting that, under the selected experimental conditions (cooling at 10 °C/min), Block-D,L and Block-L crystallizes while Ran does not show any crystallization peak. This fact evidences a strong difference in the crystallization rate of the samples. In particular, the lower crystallization temperature showed by the Block-L sample could be related to the high content of LA segments, which slow down the reorganization of the polymer segments for crystallizing.

For Block-L sample, the presence of two peaks in the first heating is related to the presence of two different kinds of crystals, but the presence of only one crystallization peak during cooling seems not compatible with this hypothesis. A second heating scan has been carried out in order to clarify this aspect (Figure 5C). In this case, the melting curves of commercial PCL and PLA are also reported for comparison. All thermal properties of these materials, such as crystallization temperature (Tc), melting temperature (Tm), melting enthalpy (Δ*Hf*), crystallization enthalpy (ΔHc), and crystallinity (*Xc*), are reported in Table 3.

When compared with both homo-polymers, the interpretation of the melting curves of the block copolymers becomes clearer. The correspondence between the various melting peaks allows their attribution. The melting peaks located at low temperatures (30–60 °C) can be attributed to the crystalline domains formed by the ε-CL segments, while those at high temperatures (150–160 °C) to the crystalline domains formed by the LA segments. For example, during the second heating scan, the Block-D,L sample showed a single melting peak with a maximum at about 52 °C and a Δ*Hf* = 34 J/g. The latter enthalpy value is very close to that recorded during the crystallization phase in cooling. This excludes the possibility of cold crystallization and allows us to assign this melting peak to the crystals formed by the ε-CL segments. Compared to the melting peak of the commercial PCL (Δ*Hf* = 87 J/g), the melting peak of the Block-D,L is about half and is placed at slightly lower temperatures. It evidently suggests less regular crystalline structures if compared to that formed by the homo-polymer. For the Block-L sample, upon cooling ε-CL segments crystallize at 17 °C with an enthalpy value of 36 J/g. The melting peak associated with this phase has an enthalpy value (Δ*Hf* = 37 J/g) larger than that showed by the Block-D,L peak. In this case, the presence of LA segments seems to slightly favor crystallization at low temperatures.

The Block-L sample exhibits also a clear exothermic peak at temperatures immediately above the first melting peak (above 70 °C). Probably, the melting of crystals with a low melting temperature allows the cold crystallization of the LA segments. The crystallinity formed at this temperature, when heated, melt at temperatures close to those characteristic of PLA (159 °C) with an enthalpy value of 15 J/g. The formation of a second crystal phase inside the Block-L material suggests the possibility to lock the polymer chains into a certain temporary shape. Both thermal transitions contribute to the fixation of the temporary shape.

### 3.4. Rheological Analysis

**Figure 6:**
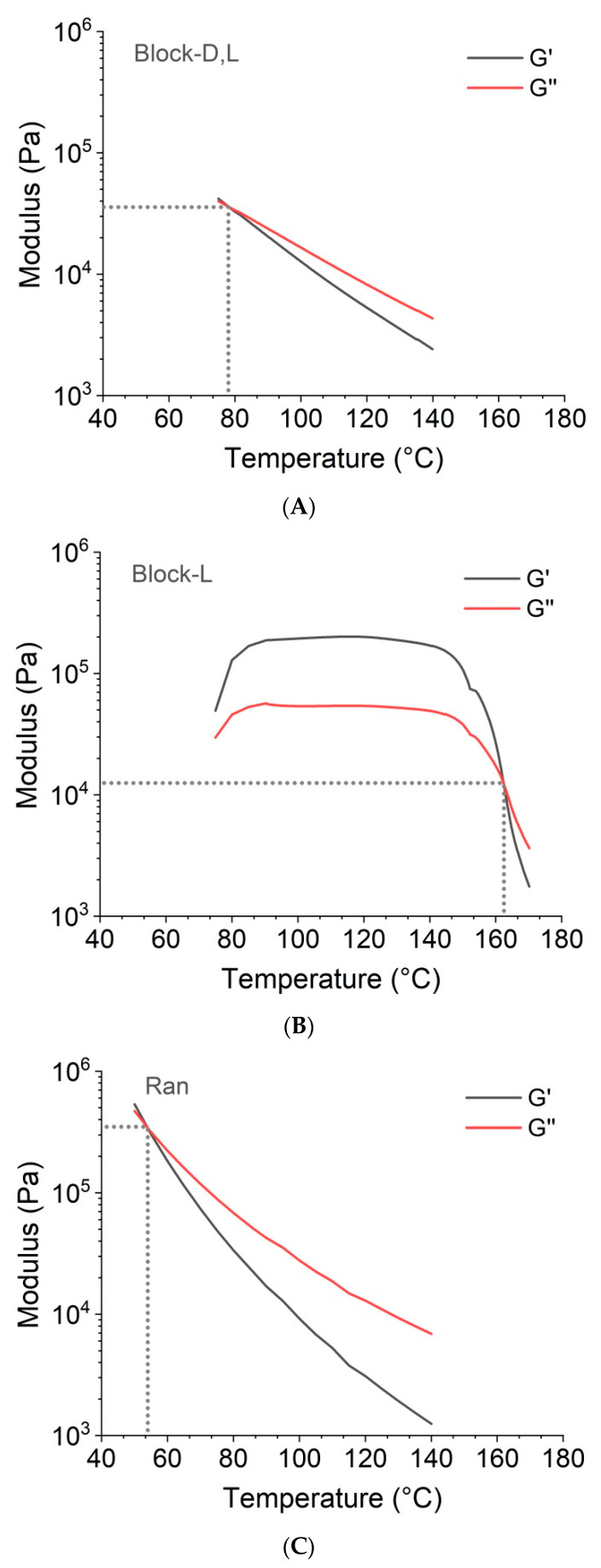
Evolution of the elastic (G′) and viscous (G″) moduli as a function of temperature (Isochronal tests) carried out at 1 Hz of frequency and 0.1% of strain for (**A**) Block-D,L, (**B**) Block-L and (**C**) Ran samples.

The thermo-rheological response of the copolymer was firstly analyzed with isochronal measurements. Figure 6 shows the evolution of the elastic (G′) and viscous (G″) moduli in a temperature sweep experiments. As temperature increases, a gradual transition is noticed from a solid-like behavior (G′ > G″) to a liquid-like behavior (G′ < G″) [47]. The temperatures at which the moduli cross (G′ = G″) are completely different in the three copolymers. These transitions are associated to the melting of the physical crosslinking formed by the crystals present in the matrix. As show by the previous DSC results, these temperatures correspond to the melting peak temperatures of the crystalline phases Figure 6. For example, the Block-D,L shows a melting temperature at T_m_ = 59 °C, which correspond to the temperature crossover of the moduli observed at T = 77 °C.

Block-L sample show an increase in both the elastic and viscous in the temperature range between 60 and 90 °C. It is probably due to the cold crystallization of the LA segments (Figure 5C). Since the low level of crystallinity formed in the sample, the increase in storage modulus is less than one order of magnitude.

The effect of crystallization on the mechanical properties of thermoplastic polymers has been evaluated by numerous works of literature [47]. For example, the microstructure developed during PLA crystallization was found to have a profound effect on the final properties [48]. Experimental results found with random and block copolymers of 2-ethyl-2-oxazoline and 2-nonyl-2-oxazoline showed a gradual transition from viscous to elastic behavior as crystallinity increased [49]. In the case of PCL-PLA copolymers, since both PCL and PLLA are crystalline blocks, the crystallization of each block is affected by the respective monomer distributions [50]. PLA can have both a positive and a negative effect on the crystallinity of PCL. In this case, PLA is solid at the crystallization temperatures of PCL and it can act both as a nucleating agent and as an obstacle to the formation of PCL crystals [51]. Conversely, the crystallization of PLA is negatively affected as the PCL content increases [52]. In fact, at the crystallization temperatures of PLA the PCL is melt and acts as a diluent for the PLA.

After the initial increase, both modules stabilize on a constant value over about 50 °C. In this temperature range (95–145 °C) the elastic modulus is about one order higher than the viscous modulus. It demonstrates a stable and clear elastic character of the materials. At temperatures higher than 150 °C, there is a significant modulus drop. This decrease in the modulus is evidently associates with the melting of the crystal formed by the LA segments (159 °C). These crystals are able to form an efficient crosslinking network that, upon melting, determines a drop in the elastic modulus larger than two orders of magnitude. The crossover point is located at 162 °C, which is in agreement with the DSC results.

**Figure 7:**
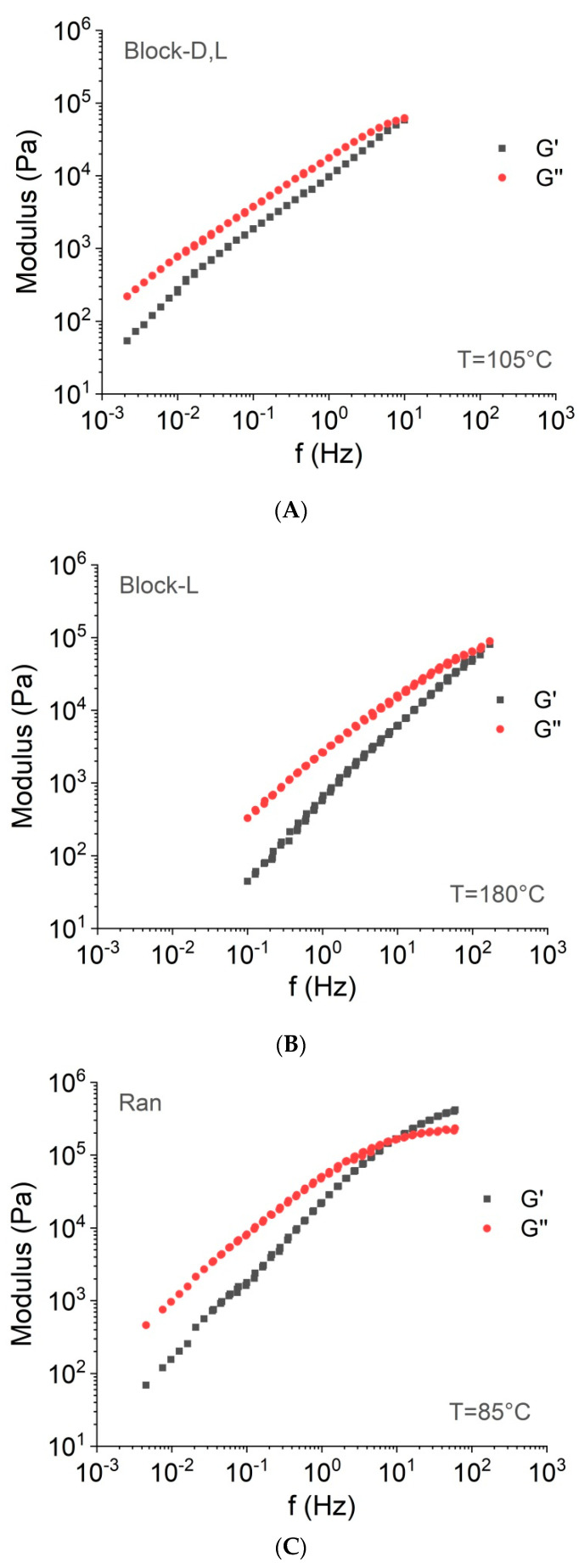
Evolution of the elastic (G′) and viscous (G″) moduli as a function of frequency carried out at reference temperature and 0.1% of strain for (**A**) Block-D,L, (**B**) Block-L and (**C**) Ran samples.

Isothermal frequency sweeps at high temperatures can bring additional information on the viscoelastic behavior of the sample [53]. For comparison, a different reference temperature (Tr) was chosen for the samples. A temperature 30 °C higher than the crossover temperature of the three copolymers was chosen. The master curve of G′ and G″ as a function of frequency are presented in Figure 7. The thermo-rheological simplicity of the systems at the reference temperature was confirmed by the good time–temperature superposition of G′ and G″ (see Figure 7). For all samples, G′ and G″ increased with increasing frequency. The elastic modulus curve lies below the viscous modulus curve, indicating a liquid type viscoelastic behavior.

In any case, the behavior of the dynamic modules of the samples is quite similar. The small differences are due to the different molar masses distributions. In general, the cross over point (where G′ = G″) occurs at lower frequency in samples with higher molar mass and widest molecular mass distribution. In agreement with the data show in Table 1, the sample with the higher molar mass (Ran) shows the crossover at lower frequency.

### 3.5. Shape Memory Test

**Figure 8:**
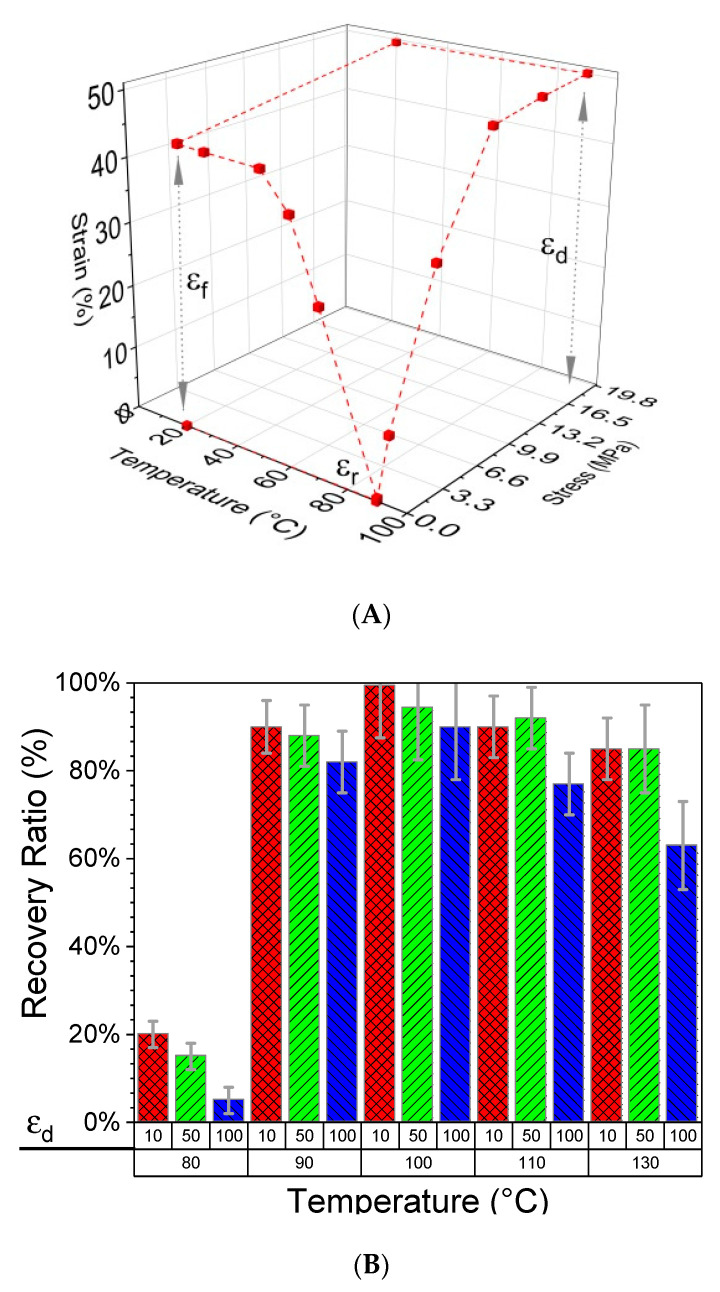
Shape memory tests: (**A**) thermomechanical cycle of Block-L sample (**B**) Recovery ratio as function of temperature and initial strain.

The shape-memory properties have been quantified by cyclic thermo-mechanical tests [43,54]. Experimental results for sample Block-L are shown in Figure 8. Only this sample demonstrates the ability to fix the temporary shape and to recover its initial shape.

Loading are achieved by drawing at temperature higher than the cold crystallization of LA segments (above 80 °C), whereas the strain recovery took place upon heating above the melting temperature of the ε-CL segments (above 60 °C).

As show by the thermo-rheological isochronal tests, the crystalline domains formed by the LA segments are enough to confer the necessary elastic character to the material, whereas the ε-CL segments domain are sufficient to stabilize the deformation during the fixation procedure.

The relationship between polymer deformation or phase transition and properties is quite similar to the relationship existing in some inorganic materials [55,56].

Experimental results of tests carried out by changing the deformation temperature and the deformation strain are showed in Figure 8B. Rr is found strongly depend on both the deformation temperature and the initial strain (εd). In the best case, the Rr ratio is high than 99%, whereas the Rf ratio values reached 85%. Repeated experiments on the sample indicate that, for the chosen conditions, only small differences are found between the thermomechanical cycles. These results demonstrate the excellent ability of Block-L materials to recover their initial shape.

## 4. Conclusions

In this work, degradable block and random PLA/PCL copolymers were synthetized and fully characterized. The designed copolymers had similar LA:CL monomer ratio, while they differ in the microstructure. The thermal degradation of the sample was dependent on the LA/ε-CL ratio. In particular, both the onset temperature (T5%) and the degradation temperature (Td) were improved by increasing the ε-CL content. DSC results showed different crystallization behaviors of the copolymers. The random (Ran) sample does not crystallize at all in the selected conditions, whereas the Block-L poly(L-lactide-*block*-ε-caprolactone) sample showed the co-existence of two crystalline phases associated with two melting peaks. The phase behavior and the molecular structure of the samples were studied by means of dynamic viscoelastic measurements. These tests have showed the relation between the mechanical moduli and the temperatures at constant frequency. The transition between solid-like to a liquid-like behavior was characterized in all samples. Block-L sample showed a complex behavior with a stability region were the sample show a predominant elastic character. At higher temperatures, the copolymers showed the typical behavior of a single phase polymer system, with a thermo-rheological simple behavior.

Block-L sample showed very good shape-memory properties in a wide range of temperature and strain. These results indicate the possibility to design biodegradable multifunctional copolymers by a low cost and an easy scalable process. These obtained materials, which show a wide range of mechanical behavior, can cover a wide range of applications, from biomedical to packaging applications.

In conclusion, this work revealed that the morphology and the mechanical properties of block and random copolymers of lactide and ε-caprolactone could be well controlled by changing the block length and solidification conditions. Such control is important for the development of efficient shape memory polymer.

## Figures and Tables

**Table 1:** Copolymerization of caprolactone and lactides catalyzed by complex I.

Run ^a^	LA:CL (in the Feed)	Yield ^b^	Copolymer Composition ^c^			Mn ^d^	*Ð* ^d^
			**D,L-LA**	**L-LA**	**ɛ-CL**		
	(mol:mol)	(*w*/*w*%)	(mol%)	(mol%)	(mol%)	(kDa)	
1 (Block-D,L)	26:74	76	27	-	73	43.4	1.4
2 (Block-L)	26:74	88	-	29	71	54.7	1.4
3 (Ran)	26:74	90	-	22	78	71.8	1.5

^a^ Reaction conditions: time: 96 h for 1 and2; 72 h for 3; temperature 90 °C, toluene = 8.5 mL. L-lactide 0.70 g, ε-caprolactone 1.5 mL, complex (I) 20 mg, BnOH: 0.5 mL (solution 0.1 M in toluene). ^b^ Determined by weighting the final copolymer. ^c^ Determined by ^1^H NMR. ^d^ Determined by GPC in THF solvent at 30 °C.

**Table 2:** TGA results of the analyzed samples.

Sample	T5% (°C)	Td1 (°C)	Td2 (°C)	ΔT1 (°C)	ΔT2 (°C)
Dev.Std.	(±1 °C)	(±1 °C)	(±1 °C)	(±2 °C)	(±2 °C)
PLA	312	355		43	
Block-D,L	285	319	368	34	83
Block-L	295	320	374	25	79
Ran	315	355	391	40	76
PCL	355		393		38

**Table 3:** Thermal properties of the analyzed materials.

	Tm 1st Heating	Δ*Hf* 1st Heating	Tc Cooling	ΔHc Cooling	Tc 2nd Heating	ΔHc 2nd Heating	Tm 2nd Heating	Δ*Hf* 2nd Heating	*Xc*(PLA) 2nd Heating	*Xc*(PCL) 2nd Heating
	°C	J/g	°C	J/g	°C	J/g	°C	J/g	-	-
Dev. Std.	(±0.1)	(±0.05)	(±0.1)	(±0.05)	(±0.1)	(±0.05)	(±0.1)	(±0.05)	(±3)	(±3)
Block-D,L	59.3	50.17	24.7	34.18			52.3	34.13		24%
Block-L	56.3	50.88	17.4	36.66			51.9	37.09		27%
	161.3	15.85			71.1	10.59	159.6	15.31	11%	16%
Ran	40.6	16.45	-	-			-	-	-	-
PCL							60	87.88		63%
PLA			78				162	51.15		55%

## Data Availability

Not applicable.
